# Severe infections after teeth removal – are we doing enough in preventing them?

**DOI:** 10.4317/jced.59314

**Published:** 2022-03-01

**Authors:** Niina Rautaporras, Johanna Uittamo, Jussi Furuholm, Johanna Snäll

**Affiliations:** 1 DDS, Department of Oral and Maxillofacial Diseases, University of Helsinki and Helsinki University Hospital, Helsinki, Finland; 2 MD, DDS, PhD, Department of Oral and Maxillofacial Diseases, University of Helsinki and Helsinki University Hospital, Helsinki, Finland

## Abstract

**Background:**

The present study clarified features and prehospital care in patients with severe infection after teeth removal.

**Material and Methods:**

Patients who were hospitalized for infection following teeth removal were included in this study. Background variables and infection severity parameters were compared between patients who underwent elective and acute teeth removal prior to hospitalization. Additionally, associations of these variables with antibiotic use were evaluated.

**Results:**

Of the 118 patients included in the study, teeth removal was due to acute infection in 64% and removal was elective in 36%. The time span from teeth removal to hospitalization varied considerably (from <1 day to 205 days). The variation was significantly greater in patients with preceding acute removal than those with elective removal (*P*=0.030). Smoking was significantly associated with acute teeth removal (*P*<0.001). Length of hospital stay (LOHS) was a day longer in the elective group (*P*=0.017). Overall, 70% of patients received antibiotics prior to hospitalization. There was a significant association between removal type and antibiotic use (*P*=0.005); antibiotic use was less common in elective teeth removal patients. Immunocompromised patients received antibiotic prophylaxis significantly more often than non-immunocompromised patients (*P*=0.003). LOHS was significantly associated with prehospital antibiotic use (*P*=0.035). LOHS was a day longer in patients who had not received antibiotics than in other patients.

**Conclusions:**

Severe infection can develop with a long delay after acute teeth removal. More attention should be paid to preceding symptoms and early effective treatment of these infections. A more precise timing of antibiotic use could reduce severe postoperative infections in elective teeth removal.

** Key words:**Odontogenic infection, teeth removal, antibiotic use, prophylaxis, postoperative infection.

## Introduction

Odontogenic infections (OI) are common infections in the head and neck region (1,2). OIs may be fatal if the infection spreads to deep neck spaces and causes acute airway obstruction, or if the infection leads to complications such as endocarditis or sepsis (3-5). Postoperative infections account for a significant proportion of hospitalizations due to OIs. Previous studies have shown that 12–51% of severe OIs emerge after teeth removal (6-8), especially removal of the mandibular third molar. Despite the high proportion of postoperative infections in severe OIs, the circumstances of these infections have rarely been studied.

OIs have become more severe in recent decades, as indicated by increasing inflammatory parameters and increased proportions of patients requiring intensive care unit (ICU) treatment (9,10). The use of antibiotics in dentistry is increasing (11,12), although there are no uniform international prescribing standards (13). The total benefit of antibiotic use in teeth removals, especially antibiotic prophylaxis, has been widely discussed and many studies have reported conflicting results (14-17). In addition, the clinical status of the removed tooth is often not considered when assessing the benefit of antibiotic therapy (16).

The purpose of the present study was to clarify infection features and prehospital care in patients with severe infection after teeth removal. We hypothesized that there are differences between background variables for infection development, which may assist clinicians in providing preventive care for severe postoperative OIs. 

## Material and Methods

-Study design and inclusion and exclusion criteria

A retrospective cohort study was conducted in the Töölö Hospital Emergency Department of Helsinki University Hospital, which between 2015 and 2018 had a catchment area of 1.6 million inhabitants. Patients who were treated in the oral and maxillofacial surgery’s emergency service and hospitalized for infection following teeth removal were included in this study. Patients with a non-odontogenic focus or unclear focus were excluded. Patients with prehospital teeth removal at the same appointment as admission were also excluded.

-Study variables

Patients were divided into the following two groups according to teeth removal type: patients with elective teeth removal and those who underwent teeth removal in a symptomatic acute state. Symptoms of acute state included pain, swelling, elevated body temperature, trismus, pus, and abscess formation. Teeth removal performed due to asymptomatic chronic infections were included in the elective group.

Prehospital and infection severity variables were assessed to compare differences between teeth removal groups.

Prehospital variables included age, sex, current smoking, current alcohol and/or drug abuse, immunocompromised state, site of infection, and time from teeth removal to hospitalization. Alcohol abuse was defined according to Finnish Current Care Guidelines (18) (FCCG) for heavy alcohol consumption limits, which are >12 doses per week in women and >23 doses in men.

Infection severity variables included C-reactive protein level, white blood cell count, and tympanic body temperature at hospital admission, ICU treatment, and length of hospital stay (LOHS).

Previous study variables were examined for antibiotic use prior to hospitalization. Patients were divided in the following three groups according to prehospital antibiotic use: 1) patients who had received antibiotics only preoperatively or pre- and postoperatively (ABPP), 2) those who had antibiotics only postoperatively (ABOP), and 3) those who had not received any antibiotics before hospitalization (noAB). The preoperative antibiotics in the ABPP group included antibiotic prophylaxis and an ongoing course of antibiotics targeted to oral bacteria at the time of teeth removal. Patients were included in the ABOP group if the course was started during the procedure at a lower dose than prophylaxis. In the FCCG, antibiotic prophylaxis in dentistry is defined as the administration of a systemic antimicrobial agent 30–90 minutes preoperatively.

Additionally, we gathered information about bacterial findings in pus samples.

-Statistical analysis

We used statistical software package IBM SPSS for Macintosh (version 26.0, IBM Corp., Armonk, N.Y. USA) to analyse patient data extracted from electronic health records. Categorical variables were cross-tabulated and associations between the study groups were evaluated with Pearson’s chi-squared test or Fisher’s exact test if expected cell values were below 5. Differences in continuous variables were assessed with Student’s t test or analysis of variance for normally distributed parameters, and with Mann-Whitney U test or Kruskal-Wallis H test for other continuous variables. Pairwise post-hoc analyses were conducted with Z test for categorical and Dunn’s procedure for continuous variables with Bonferroni correction. We considered *P*-values <0.05 as statistically significant throughout the study. 

## Results

A total of 335 patients with maxillofacial infection were hospitalized. Of these, 217 patients were excluded from the final analysis for the following reasons: focus tooth was not removed prior to hospitalization (180 patients), non-odontogenic or unclear focus (32 patients), and focus tooth was removed just prior to hospital admission by the referring dentist (5 patients). Thus, 118 patients were included in the analysis.

Sex distribution was even (males, n=57; females, n=61). Patient age ranged from 18 to 92 (mean 44, median 43) years. Thirty-four (29%) patients were treated in the ICU. Teeth removal was performed electively in 36% of patients and in acute state in 64%.

Time from teeth removal to hospitalization ranged from <1 to 205 (mean 8.5, median 3) days. The timeline between teeth removal and hospital care varied widely among patients with previous teeth removal at acute state compared with an elective procedure (*P*=0.030).

Smoking and alcohol and/or drug abuse was relatively low; 29 (25%) patients were current smokers and only 7 (6%) abused alcohol and/or drugs. Only 5% of elective-group patients were smokers, whereas the percentage of smokers in the acute group was seven-fold higher (36%) (*P*<.001) (Table 1).

The mandible was the infection site more often than the maxilla (85% vs. 15%). The most common removed tooth prior to hospitalization was mandibular other than third molar (45%) followed by the mandibular third molar (40%, 47 of 118). Mandibular third molars were evenly distributed between acute and elective groups (55% and 45%), whereas most of the other mandibular teeth (81%) and maxillary teeth (67%) were removed while acutely infected (*P*=0.001) (Table 1).

**Table 1:**
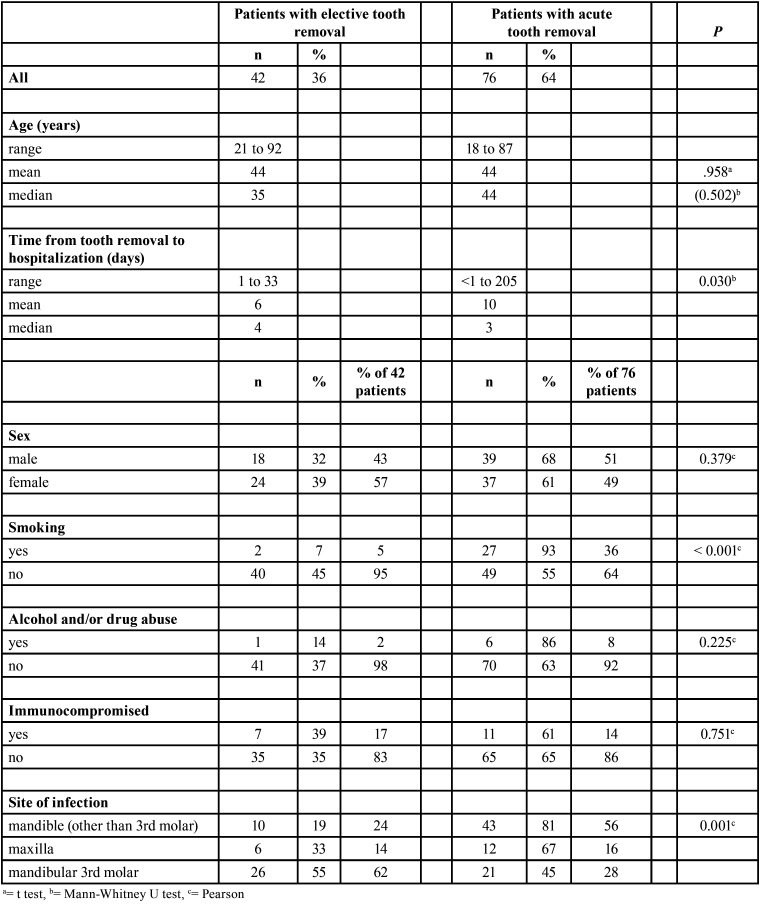
Associations between prehospital variables and type of tooth removal in 118 patients with postoperative infection.

LOHS was a day longer in the elective group than in the acute group (*P*=0.017). Prehospital antibiotic use also influenced LOHS (*P*=0.035). LOHS was a day longer in noAB-group patients (*P*=0.035). All infection parameters at hospital admission were slightly higher in the elective group and in the noAB group, although this was not statistically significant ( Table 2- Table 4).

**Table 2:**
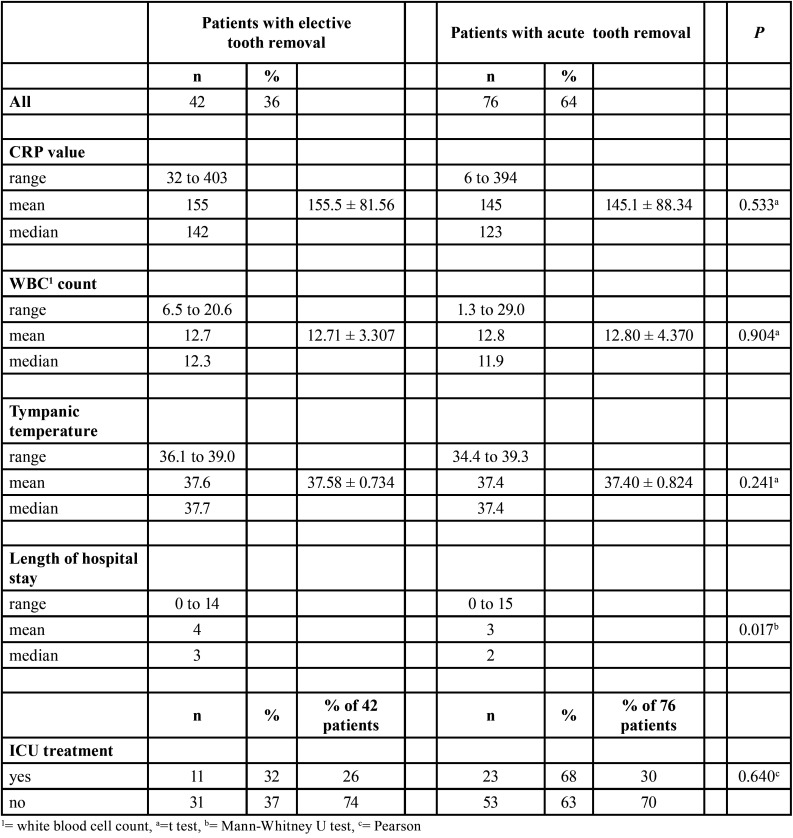
Associations between infection severity variables at hospital admission and type of tooth removal in 118 patients.

**Table 3:**
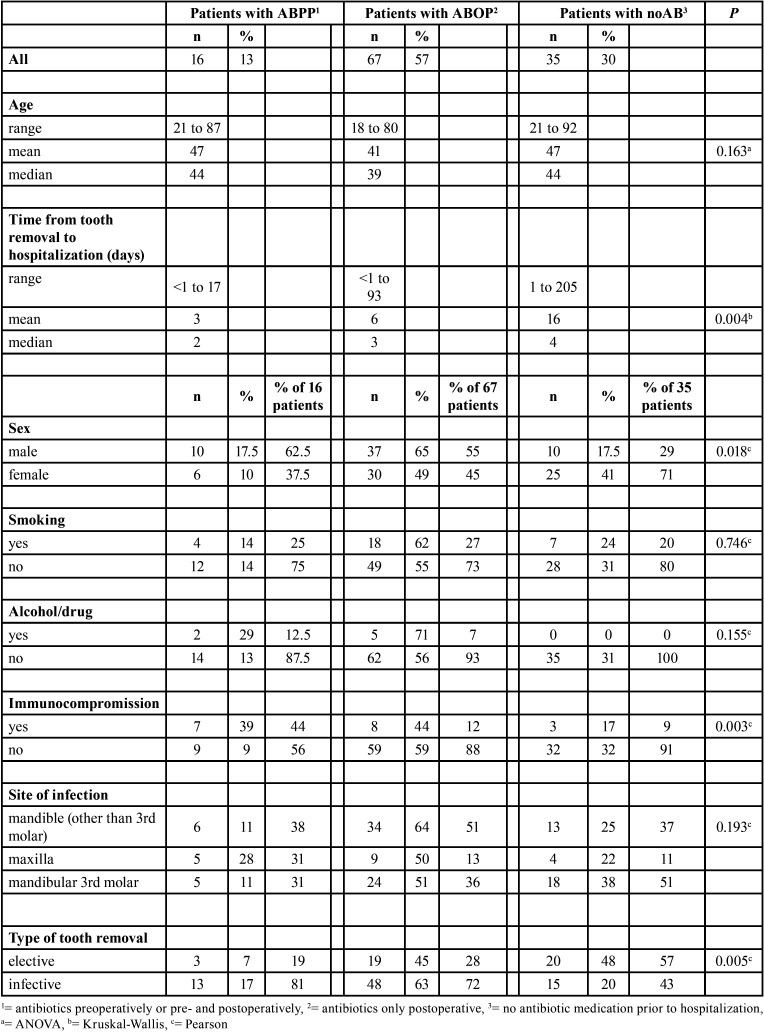
Associations between prehospital variables, type of tooth removal and antibiotic use in 118 patients.

**Table 4:**
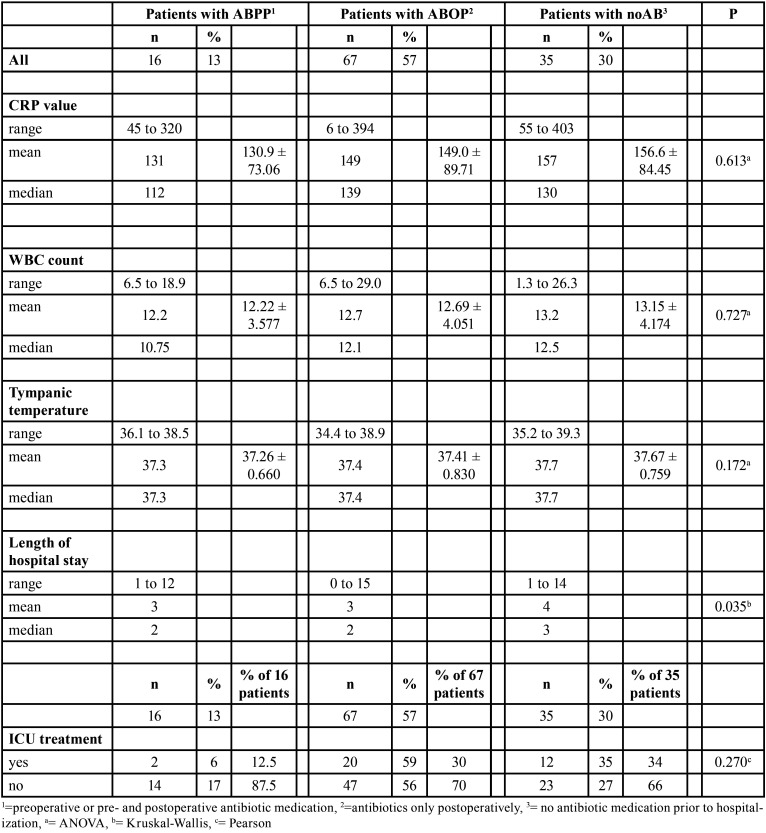
Associations between infection severity variables at hospital admission and prehospital antibiotic use in 118 patients.

Antibiotics were given to 83 (70%) patients prior to hospitalization; 13% received antibiotics prophylactically before teeth removal (Table 3). Of all patients, 30% did not receive any antibiotics before hospital admission. Antibiotic use was significantly distributed between type of removal (*P*=0.005). Almost half (48%) of patients with elective teeth removal and one fifth (20%) in the acute teeth removal group did not receive any antibiotics prior to hospitalization. A large variation in timing of teeth removal was also observed regarding use of antibiotics (*P*=0.004). The time span from teeth removal to hospitalization was longest in the noAB group.

Only 18 (15%) patients were immunocompromised. Of these patients, 10 had diabetes. The patient’s immunocompromised state affected the use of antibiotics (*P*=0.003); immunocompromised patients received ABPP more often (39%) than non-immunocompromised patients (9%) (Table 3). The use of antibiotics also differed between sexes (*P*=0.018); males received ABOP more often whereas patients in the noAB group were more commonly females.

The bacterial findings were overall consistent in both teeth removal groups. While the most common bacteria cultured from pus samples was Viridans Group Streptococcus, in general these infections were predominantly caused by mixed flora of anaerobic and aerobic bacteria.

## Discussion

A tooth removal often precedes severe OI, but little has been reported on the background of this OI subtype. The purpose of the present study was to clarify infection features and prehospital care in patients with severe infection after teeth removal. We confirmed our hypothesis that there are clinically notable differences between background variables for infection severity and infection development. In 64% of the 118 patients, teeth removal was conducted for acute symptoms and removal was elective in the remaining 36%. These groups had different characteristics. The time from teeth removal to hospitalization varied widely in the acute removal group. Additionally, median LOHS was 1 day longer in patients with elective teeth removal than those with a previous acute teeth removal. Shorter hospital stay was also associated with antibiotic use prior to hospitalization.

In our study, a trend towards more severe infection in electively treated patients compared with acute teeth removal patients was observed. In addition to a significantly longer LOHS, all infection parameters were slightly higher at hospital admission in electively treated patients, even if this was not statistically significant. This can be partly explained by the finding that the lower third molar was the most commonly removed tooth in electively treated patients (62%). Additionally, chronic or subacute infection typically precedes acute OI, whereas in elective procedures the infection develops after local tissue damage. These findings raise questions on optimal prophylactic antibiotic use, circumstances during teeth removal, and local conditions predisposing to postoperative infections.

Seventy percent of patients received antibiotics prior to hospitalization. Infection parameters were slightly higher in the noAB group than in patients who had used antibiotics (Table 4) and LOHS was 1 day longer in the noAB group. Prescribing of excess antibiotics for extended durations is common and prescription patterns lack consistency (13,19-21). For example, the rate of antibiotic prescription after teeth removal in South Korea in 2011-2015 was 82% (22). Between 1996 and 2013 in British Columbia (Canada), the amount of antibiotic prescriptions made by dentists increased by 62% (11). In contrast, from 2013 to 2015 in the United States, the number of antibiotics prescribed by dentists remained sTable and inappropriate use decreased; this may be due to narrowed indications for antibiotic use (23). Also, patients´ perceptions prefer antibiotic use after tooth removal, in the recent study made by Pérez-Amate (24) 77% of patients thought that antibiotics are necessary after tooth removal. However, the use of antibiotics should be optimally targeted.

Only 16 patients (13%) received ABPP and of these only 3 were treated electively. Thus, severe infection after elective teeth removal in a patient with ABPP is rare. Lodi *et al*. (14) stated in their Cochrane review that there is no clear evidence that timing of antibiotic administration is important in preventing complications after teeth removal. However, the review consisted primarily of studies focused on elective mandibular third molars in healthy patients. In our study, nearly two thirds of the hospitalized patients underwent acute teeth removal. The number of immunocompromised patients was low (n=18), and immunocompromised state was significantly associated with prophylactic antibiotic use (*P*=0.003). Therefore, to prevent the greatest proportion of severe OIs after teeth removal, it would be beneficial to focus on acute removals and careful treatment of local infection spread in healthy patients.

**Figure 1:**
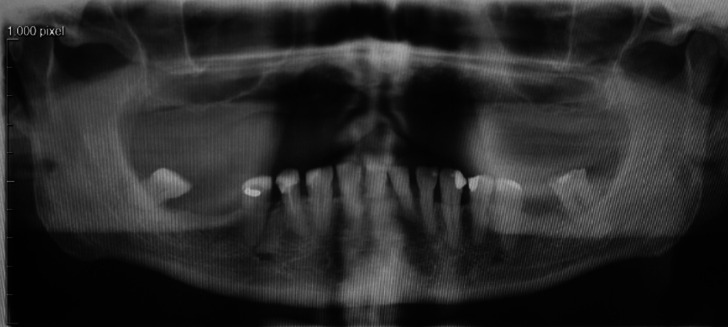
A non-smoking 72-year-old man applied to a dentist due to a week-long toothache. The patient had medication for hypertension. Symptoms localized to the completely erupted lower right 3rd molar with local periodontal infection. The patient had body temperature of 38 degrees, but no other symptoms of generalized infection. The tooth was removed by the dentist and the patient received a postoperative antibiotic course.

**Figure 2:**
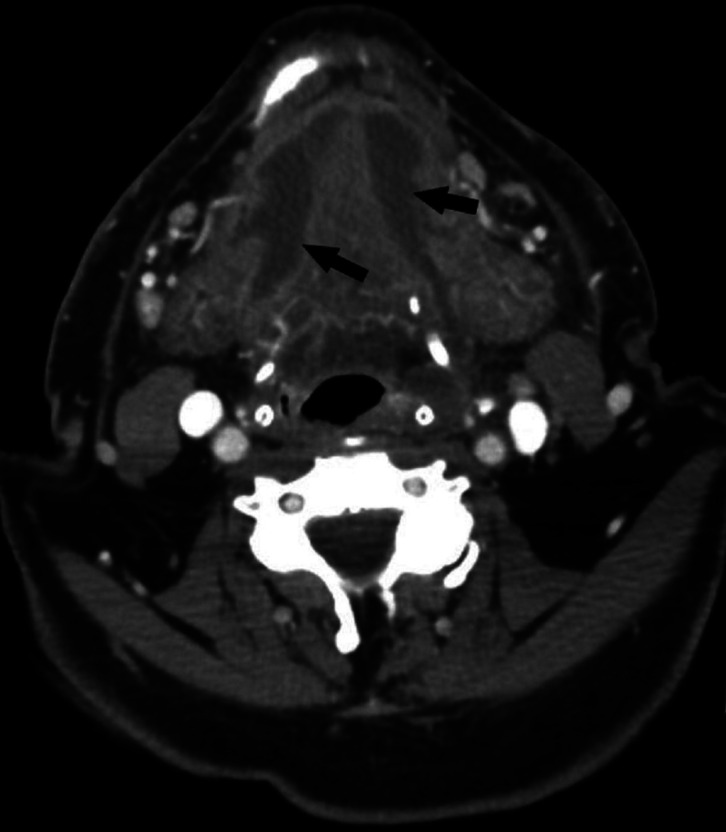
Two days later the patient reappeared to the dentist due to difficulties in swallowing and mouth opening. Typical findings of Ludwig’s angina were observed: mouth floor swelling, difficulties in speaking and swallowing and limited mouth opening. The dentist referred the patient to hospital. Features of severe infection were detected also in infection parameters: Body temperature was 38.5, C-reactive protein level (CRP) was 342 mg/l and white blood cell count was 19.2 E9/l. Computer tomography images (see also Fig 3.) confirmed the clinical diagnose of bilateral abscess which had spread from the mandibular third molar area (arrows). The airway was also restricted.

**Figure 3:**
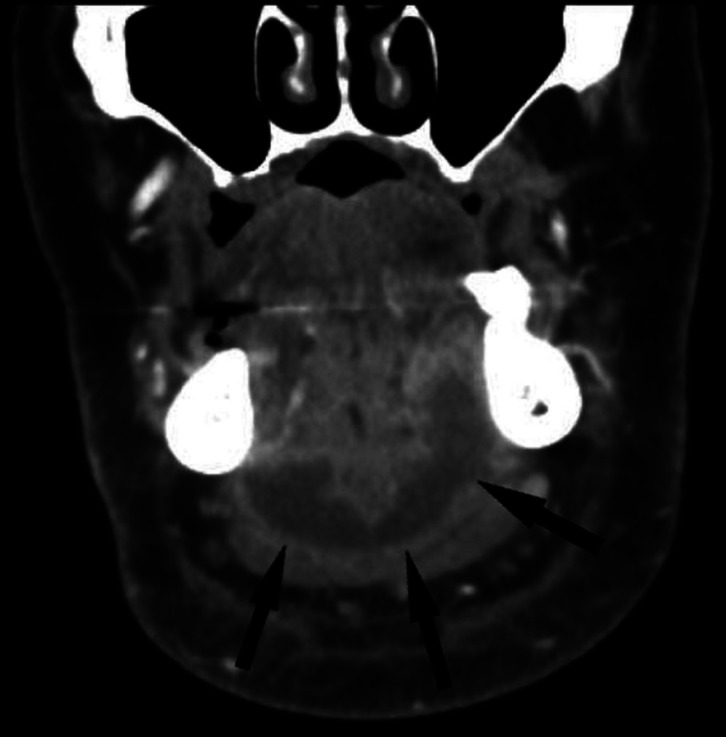
On both sides in the sublingual space and on top of and partly inside the muscles of the mouth floor, broad plate-like abscess (arrows). The abcess was incised, drained and extraoral drains were placed under general anesthesia by maxillofacial surgeons. Patient was treated in the intensive care unit for 5 days because of extensive swelling and septic symptoms.

Cervino *et al*. (16) stated that the clinical status of the third molar is often not considered when studying the use and benefits of antibiotic prophylaxis. Thus, local circumstances are often underestimated. In addition, our previous study revealed that although half of the OI patients admitted to hospital had preceding health care contact for infection symptoms (25). The most commonly received treatment was solely antibiotics. The median time range from teeth removal to hospitalization in the present study was 3 days (mean 8.5). However, the wide time range in the acute removal group (even up to months) confirms that hospitalized OI patients can have symptoms for a surprisingly long time. Thus, instead of considering antimicrobial therapy exclusively, clinicians should focus more on clinical infection signs, early detection of infection, and comprehensive local OI care (Figs. 1-3).

In the current study, smokers had teeth removal significantly more often at acute state (93%), whereas only 7% were hospitalized for a preceding elective procedure. In addition to known common surgical side effects (26) and local healing in oral cavity (27-29), smoking can alter the oral microbiome (30). Considering our finding, it is possible that smoking is common among patients who visit a dentist for teeth removal at the acute state. In all, smokers are a significant group among severe OI patients. Up to 39% of hospitalized OI patients are smokers (31).

Due to the retrospective nature of our study, we were unable to systematically assess the difficulty of the teeth removals. Surgeon experience and procedure duration may also affect postoperative outcomes (16). The role of additional supportive care, such as chlorhexidine mouth rinse, could not be evaluated from the retrospective study design and this can be considered as a study limitation.

## Conclusions

Severe infections most often occur after acute-state teeth removal. The time from teeth removal to hospitalization varies considerably, even up to several months. Thus, clinicians’ knowledge of early identification and effective treatment of developing OIs should be improved to reduce most severe infections. OI characteristics after elective procedures differ from those with acute procedures; a trend towards more severe infections was observed after elective teeth removal.
